# Supplementary material for: HCV infection induces ubiquitin-dependent degradation of LATS1, inactivating the Hippo pathway and upregulating transcription of the CYR61 and CTGF genes
Source: J Gen Virol. 2026 Feb 5;107(2):002221. doi: 10.1099/jgv.0.002221 (PMC12877566; doi:10.1099/jgv.0.002221)
Supplement: Uncited Supplementary Material 1. [file jgv-107-02221-s001.pdf]

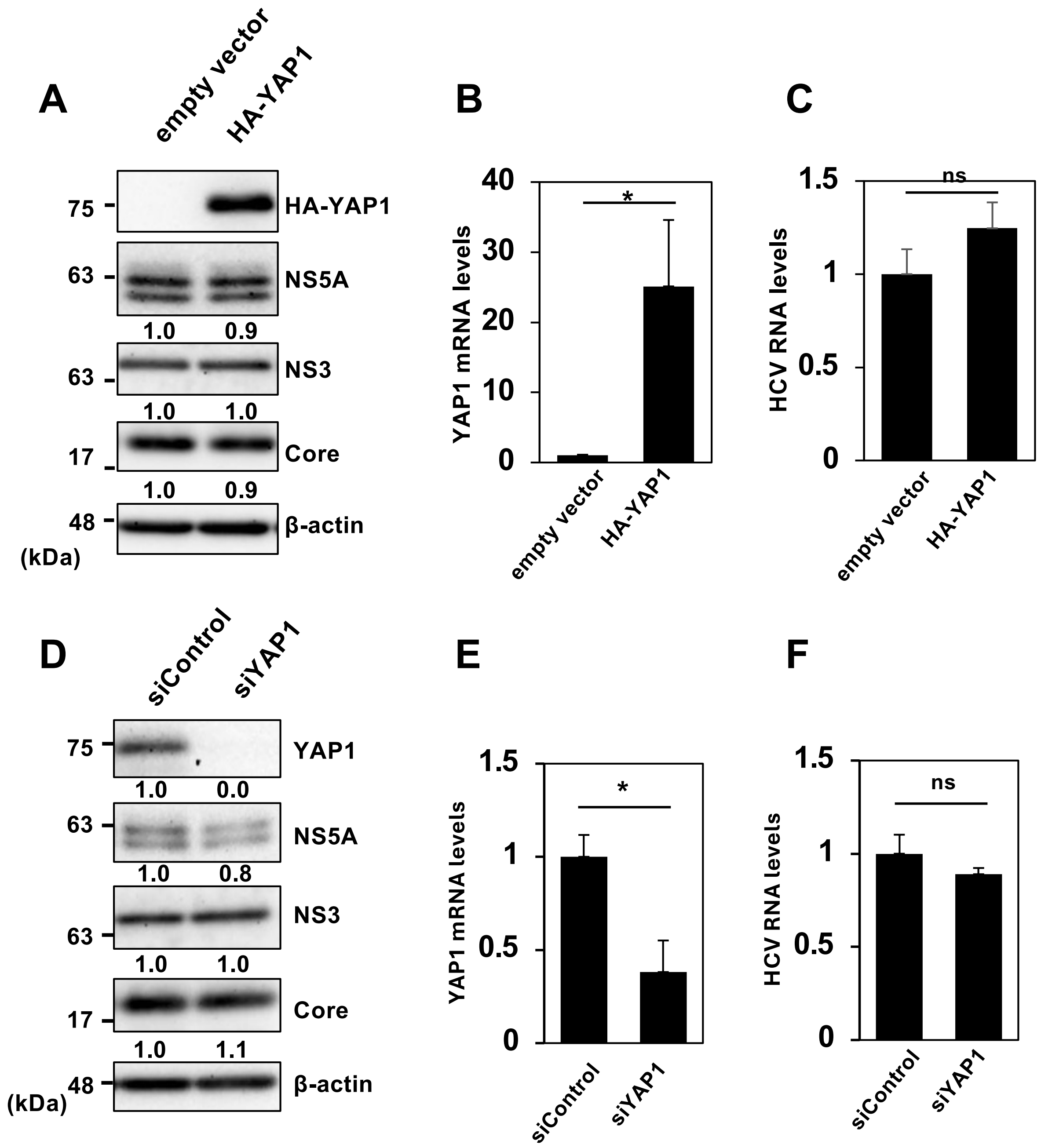

Supplementary Fig. 1 Maria et al.

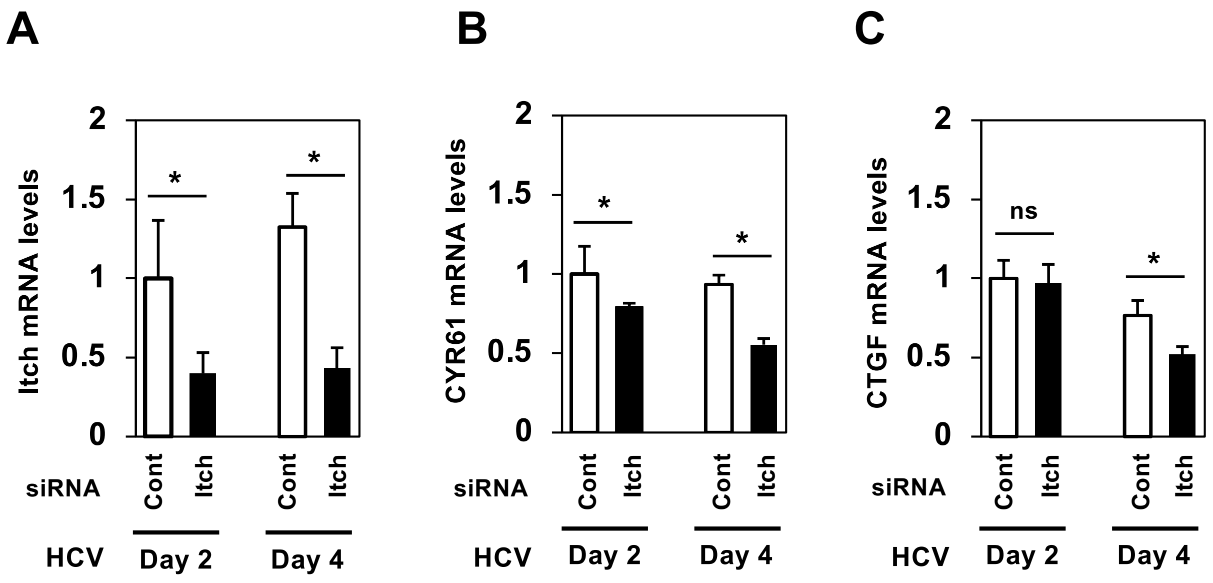

Supplementary Fig. 2 Maria et al.

Supplementary Figures

Supplementary Figure 1. Neither YAP1 overexpression nor knockdown alters HCV RNA or protein levels in Huh-7.5 cells. (A) Huh-7.5 cells were infected with HCV J6/JFH1 at an MOI of 2 and transfected with either an empty factor or pCAG-HA-YAP1. Cells were cultured and harvested at 4 days post infection (dpi), followed by immunoblotting using the indicated antibodies. β-actin levels served as a loading control. The immunoblot shown is representative of three independent experiments that yielded similar results. (B, C) Huh-7.5 cells were infected with HCV J6/JFH1 at an MOI of 2 and transfected with either empty factor or pCAG-HA-YAP1. Cells were cultured and harvested at 4 days post infection (dpi), and total cellular RNA was extracted. The mRNA levels of YAP1 (B) and HCV RNA (C) were quantified by RT-qPCR using the 2–ΔΔCt method. GAPDH mRNA levels were used for normalization. The mRNA level in the empty vector group was arbitrarily set as 1.0. Data are presented as means ± SEM from three independent experiments. The p-value < 0.05 (*) was significant. (D) Huh-7.5 cells were transfected with 48 pmol of either control siRNA or YAP1-specific siRNA. At 24 hours after siRNA-transfection, the cells were infected with HCV J6/JFH1 at a MOI of 2. Cells were harvested at 4 days post-infection and subjected to immunoblotting with the indicated antibodies. β-actin served as a loading control. The immunoblots are representative of three independent experiments that yielded similar results. (E, F) Huh-7.5 cells were transfected with 48 pmol of

either control siRNA or YAP1-specific siRNA. At 24 hours after siRNA-transfection, the cells were infected with HCV J6/JFH1 at a MOI of 2. Cells were cultured and harvested at 4 days post infection (dpi). Total cellular RNA was subsequently extracted, the mRNA levels of YAP1 (B) and HCV RNA (C) were quantified by RT-qPCR. Relative mRNA expression levels were calculated using the $2^{-\Delta\Delta Ct}$ method. To normalize the mRNA levels, GAPDH mRNA levels were used as an internal control. The mRNA level in the empty vector group was arbitrarily expressed as 1.0. Data represent means ± SEM from three independent experiments that yielded similar results; Statistical significance was assessed by Student's t-test. The p-value < 0.05 (*) was significant, compared with the controls.

Supplementary Figure 2. Effect of Itch knockdown on CYR61 and CTGF mRNA expression in HCV-infected Huh-7.5 cells. (A, B, C) Huh-7.5 cells at 1.8 × 105 cells in a 24-well plate were transfected with 24 pmol of either control siRNA or Itch siRNA. At 24 h after siRNA-transfection, the cells were infected with HCV J6/JFH1 at an MOI of 2, cultured and harvested at the indicated time points. Total RNA was extracted, and Itch, CYR61 and CTGF mRNA levels were quantified by RT-qPCR. To normalize the mRNA levels, GAPDH mRNA levels were used as an internal control. The value for day 2 mock-infected cells was arbitrarily expressed as 1.0. Data represent means ± SEM from three independent experiments that yielded similar results; Statisitical

significance was assessed by Student's t-test. The p-value < 0.05 (*) was significant, compared with the controls.
